# Patient education: A symbiotic relationship

**DOI:** 10.1177/17151635231165293

**Published:** 2023-04-12

**Authors:** Polixeni Hantjidis

**Affiliations:** University of Saskatchewan, Saskatoon, SK

Pharmacy practice includes lifelong learning from many different areas, in addition to pharmaceutical knowledge. However, some of the most valuable knowledge we can gain as pharmacists comes from our patients. Regardless of the hours we spend studying, researching and doing our best to piece together information, no one knows a condition and experience as well as the patient themselves. Patient education goes beyond educating the patient in our area of expertise—it also includes being educated by the patient. Our daily interactions and relationships with our patients are valuable opportunities to learn, appreciate and grow as health care providers. In this article, I discuss a patient interaction (with her permission) that I have experienced over the years—Anna H. I begin with Anna’s background and experience, discuss her relationship and involvement with her pharmacists and end with what I learned from her.

Anna’s struggle with her health has been a complicated one. On May 7, 2007, Anna found blisters in her mouth and headed to the emergency room. The blisters continued and got worse over months. After 6 months of struggling and deterioration in her condition, running around to different specialists and doctors and losing hope that she would be healthy again, Anna received a diagnosis of pemphigus vulgaris, a rare group of autoimmune conditions characterized by painful blisters on skin and mucous membranes. At this time, the approach to treat this condition was unknown. One of the first attempts at therapy was 60 mg of prednisone daily. Then, her side effects began. Anna experienced almost every side effect in the books for prednisone—moon-face, weight gain, severe depression, insomnia and so on. Continuing with high-dose therapy, she was additionally put on a once-weekly dose of alendronate. Anna experienced such severe bone pain by the second week that she had to be carried around the house, unable to walk on her own and screaming from pain even when sitting down. She contacted the pharmacy and was told to discontinue the medication immediately. In 2010, rituximab had officially come to the market in Canada. This was her one hope for a treatment since prednisone was the only thing keeping her alive but also ruining her life at the same time. However, she was warned about the potentially deadly effects in the first 24 hours of treatment. Anna was terrified, had 2 young kids and was not willing to risk her life on a therapy. Three years later, Anna’s condition had continued to deteriorate, as had her mental health. After 6 years of high-dose prednisone and suffering from the side effects to the point of losing hope, she agreed to try rituximab, even though she had a 12- and 17-year-old waiting for her at home. Anna’s struggle with her health has continued, and to this day, she is still fighting to manage the disease, but with the many innovations and increased education in drug therapy, she has been able to live a better life than she did 10 years ago.

Anna saw so many physicians and specialists in her struggle to find a diagnosis. It was confusing and extremely stressful for her. However, the one constant in her experience was her pharmacists. Anna was in the pharmacy more days of the month than she was not. Constantly struggling with new medications and confused about other medications, her pharmacists were always there to support and educate her. The pharmacists went above and beyond dispensing and educating her on each medication. They were a support system for her, and they were interested in her health, empathetic about her experience and always trying to figure out how to make Anna feel comfortable, safe and heard. Additionally, they were always asking her questions. Although this behaviour may seem like standard of practice, their questions were not only geared towards identifying drug therapy problems but were intended to educate themselves. It was evident that the pharmacists were listening to Anna with intent to learn from her experience and use that to provide better patient-centred care for her. Anna’s pharmacists demonstrated patient-centred care from the first prescription Anna filled. They were there for her every step of the way, constantly trying to increase their knowledge about Anna’s condition through various resources but, most important, through Anna. They tailored their care to Anna’s needs by being accessible, providing help and resources for Anna’s concerns and allowing her to be a key and active member of her own health journey.

Throughout this experience, I have learned how incredibly important it is to take the time to learn from our patients. This is important not only for us to gain knowledge as health care providers but also to be able to provide patient-centred care as effectively as possible. Patient-centred care is the key to optimal therapy for patients. Anna felt heard, cared for and safe because of the efforts of her pharmacists. Her pharmacists realized that even though they knew about the side effects of prednisone and the chances of deadly side effects from rituximab, Anna was the one experiencing this and the one who truly understood how these side effects impact quality of life.

Anna is my mom, and I was the little girl watching these pharmacists care for her as much as I did. Anna is my inspiration and the reason I value learning from patients so much. She has shown me how important it is to understand that regardless of how much we know about medications and diseases, no one truly understands a condition better than the patient experiencing it. Patient education is a symbiotic relationship. We can learn and gain from our patients just as much, or even more, as they can from us. ■

